# Controversial Issue Session: Brace treatment in infantile scoliosis: benefits

**DOI:** 10.1186/1748-7161-7-S1-O76

**Published:** 2012-01-27

**Authors:** Jean Claude de Mauroy

**Affiliations:** 1 Clinique du Parc – Lyon, France

## Background

The first prospective study of 136 children with progressive infantile scoliosis treated conservatively under the age of four years, and followed up for nine years was published in 2005 by H. Mehta. It shows that infantile scoliosis can be reversed by harnessing the vigorous growth of the infant to early treatment.

## Material and methods

In 1965, Cotrel and Morel describing the EDF plaster jacket technique stated that “in young children, it should be feasible not only to prevent further progression but above all to use the child’s growth to regress structural vertebral and thoracic deformities”. Before the child start walking, we use a plaster shell in bending correction. After walking, an underarm plaster cast with a large anterior opening, and the modified Milwaukee brace with polyethylene bars and cervical collar without hyoid support is the only brace that can manage curves in the top part of the spine. It is also the only brace that can avoid a hypoplastic thorax.

## Results

Even if IIS has the characteristic that they can be a resolving scoliosis, it must be re-emphasized that the purpose of the brace is to slow the inevitable progression of the curve, not to correct the curve. The success of conservative orthopaedic treatment depends on two factors:

1. Unlike adolescent idiopathic scoliosis, we have for infantile idiopathic scoliosis (IIS) a prognostic index: the Rib Vertebral Angle Degree (RVAD) as described by M H Mehta is the angle formed on each side between the apical thoracic vertebra and its corresponding rib.

2. IIS are often thoracolumbar curves of large radius, well accessible to bracing.

When conservative treatment is later, the goal of bracing is to allow the child to grow before a more definitive surgical procedure is done. Surgery during growth is complex and faces the technical problems of growing rods without arthrodesis.

## Conclusion

It is possible to correct definitively some IIS with an early conservative treatment. The realization of these treatments involving regularly practice of plaster casts may be difficult because of a reduced frequency of infantile scoliosis. It is one of the benefits of conservative treatment, including plaster cast before bracing.

